# Effects and factors associated with indoor residual spraying with Actellic 300 CS on malaria morbidity in Lira District, Northern Uganda

**DOI:** 10.1186/s12936-019-2681-6

**Published:** 2019-02-21

**Authors:** Abdulaziz Tugume, Fiston Muneza, Frederick Oporia, Arthur Kiconco, Christine Kihembo, Angela Nakanwagi Kisakye, Peter Nsubuga, Sekimpi Deogratias, Adoke Yeka

**Affiliations:** 1 Department of Epidemiology and Biostatistics, College of Health Sciences, School of Public Health, Makerere University, P.O.BOX, 7072, Kampala, Uganda; 2 Department of Disease Control and Environmental Health, College of Health Sciences, School of Public Health, Makerere University, Kampala, Uganda; 3 Department of Health Policy Planning and Management, Makerere University School of Public Health, Kampala, Uganda; 4 African Field Epidemiology Network, Kampala, Uganda; 5 Global Public Health Solutions, Atlanta, GA USA

**Keywords:** Malaria, Indoor residual spraying (IRS), Morbidity trends, Percentage point (pp), Test positivity rate (TPR)

## Abstract

**Background:**

Indoor residual spraying (IRS) with Actellic 300 CS was conducted in Lira District between July and August 2016. No formal assessment has been conducted to estimate the effect of spraying with Actellic 300 CS on malaria morbidity in the Ugandan settings. This study assessed malaria morbidity trends before and after IRS with Actellic 300 CS in Lira District in Northern Uganda.

**Methods:**

The study employed a mixed methods design. Malaria morbidity records from four health facilities were reviewed, focusing on 6 months before and after the IRS intervention. The outcome of interest was malaria morbidity defined as; proportion of outpatient attendance due to total malaria, proportion of outpatient attendance due to confirmed malaria and proportion of malaria case numbers confirmed by microscopy or rapid diagnostic test. Since malaria morbidity was based on count data, an ordinary Poisson regression model was used to obtain percentage point change (pp) in monthly malaria cases before and after IRS. A household survey was also conducted in 159 households to determine IRS coverage and factors associated with spraying. A modified Poisson regression model was fitted to determine factors associated with household spray status.

**Results:**

The proportion of outpatient attendance due to malaria dropped from 18.7% before spraying to 15.1% after IRS. The proportion of outpatient attendance due to confirmed malaria also dropped from 5.1% before spraying to 4.0% after the IRS intervention. There was a decreasing trend in malaria test positivity rate (TPR) for every unit increase in month after spraying. The decreasing trend in TPR was more prominent 5–6 months after the IRS intervention (Adj. pp = − 0.60, P-value = 0.015; Adj. pp = − 1.19, P-value < 0.001). The IRS coverage was estimated at 89.3%. Households of respondents who were formally employed or owned any form of business were more likely to be unsprayed; (APR = 5.81, CI 2.72–12.68); (APR = 3.84, CI 1.20–12.31), respectively.

**Conclusion:**

Coverage of IRS with Actellic 300 CS was high and was associated with a significant decline in malaria related morbidity 6 months after spraying.

## Background

Globally, 3.4 billion people are at risk of developing malaria [1]. In 2016 alone, a total of 216 million cases of malaria were recorded causing about 445,000 malaria deaths [2]. Sub-Saharan Africa is the most affected region contributing 90% of malaria cases [2]. Uganda ranks third among African countries affected by *Plasmodium falciparum*, the leading cause of malaria morbidity and mortality in sub-Saharan Africa [3]. The implementation of World Health Organization’s malaria test and treat guidelines in Uganda made it mandatory for public health facilities to produce routine data on various health outcomes including; total malaria cases diagnosed clinically, by microscopy or Rapid Diagnostic Test (RDT) at outpatient departments. Such data is collected through an integrated Health Management Information System (HMIS). According to routine malaria surveillance data, malaria is responsible for 30–50% of outpatient visits in Uganda [4].

Scaling-up of indoor residual spraying (IRS) coverage in sub-Saharan Africa has proven to be successful in reducing malaria transmission in different epidemiological settings [5, 6]. However, for IRS to be effective, it is recommended that more than 85% of households within the targeted communities that are at risk of malaria are sprayed [7]. The spread of pyrethroids and carbamates resistance in malaria vectors has influenced malaria control programmes to implement a rotational system of insecticide use in an effort to mitigate insecticide resistance [8–10].

Between 2006 and 2014, the Uganda IRS programme conducted several rounds of IRS and originally targeted only 10 malaria epidemic prone districts in Northern Uganda [11]. Due to the promising results of IRS both in terms of coverage and impact, its implementation was scaled-up to cover other 14 new districts of Northern and Eastern Uganda [12]. The Uganda IRS project Phase II chose Actellic 300 CS for IRS in the new IRS districts following reports of mosquito resistance to the previously used insecticides and those used to treat bed nets [13].

The United States President’s Malaria Initiative (PMI) and other partner organisations recommend use of Actellic 300 CS, for residual control of mosquitoes and other public health pests [10]. The organophosphate formulation is believed to provide a prolonged residual protection up to 1 year [14, 15].

Between July and August 2016, Abt Associates implemented IRS phase II in Lira District switching insecticides from bendiocarb to Actellic 300 CS [16]. Actellic 300 CS is a new insecticide that was approved by the World Health Organization Insecticide Evaluation Scheme (WHOPES) in 2013 and was introduced in Uganda in 2016 [17]. There is limited information about the effect of Actellic 300 CS on malaria control in a high transmission setting in Uganda. This study sought to describe trends in malaria outpatient attendance 6 months before and after the IRS intervention so as to estimate the effect of the new insecticide used in the Uganda IRS programme. Additionally, the study aimed to assess IRS coverage and factors associated with IRS using Actellic 300 CS on malaria morbidity in Lira District.

## Methods

### Study design

This was a mixed methods study that employed both quantitative and qualitative approaches of data collection. A retrospective review of medical records before and after the implementation of one round of IRS using Actellic 300 CS was conducted. Additionally, a household survey was conducted to assess the IRS coverage measured as ‘household spray status’ and factors associated with IRS.

### Study setting

The study was carried out in Lira District, a high malaria transmission setting in Northern Uganda. Data collection was conducted in June 2017, 12 months after spraying with Actellic 300 CS. The implementation of IRS with Actellic 300 CS in the study area took place between July and August 2016. Data collection was conducted at the time when no new malaria control intervention had been implemented in the district. At the time of the study, the district population was estimated at 408,043 with a growth rate of 2.8 per annum and about 89,133 households in 2014 [18]. Malaria transmission in Northern Uganda is persistent throughout the year with two peaks, usually after the rainy seasons [4]. According to Uganda National Meteorological Authority, Lira District experiences two major rainfall seasons from March–May and September–December [19]. The district has 30 health facilities both public and private not for profit (PNFPs) [20]. This study analysed monthly HMIS data from four high volume facilities. Specifically, the following health facilities were included in this study: Amach Health Centre IV, Ogur Health Centre IV, PAG Health Centre IV and Lira Regional Referral Hospital).

### Description of the Health Management Information System in Uganda

The Uganda Ministry of Health has an integrated Health Management Information System (HMIS) in which health facilities collect routine data on various health indicators using standardized HMIS forms. The data is collected during routine patient care and aggregated to compile monthly reports at health facility level which are submitted to the District Health Office. The HMIS-105 forms are universally used to compile health facility monthly reports. At the District Health Office, the HMIS-105 monthly data are uploaded on to the electronic version of the district health management information system (DHIS2). The hard copies of the monthly reports are archived for future reference and can be accessed on request. This study abstracted and analysed retrospective HMIS-105 paper data at four outpatient facilities, focusing on a 6-month period before and after the implementation of IRS with Actellic 300 CS.

### Study participants

The study population consisted of households from randomly selected parishes, key informants and HMIS-105 paper reports. From each selected household, one participant preferably a household head was interviewed. Key informants were purposively sampled from selected parishes and comprised of; village chairpersons, parish councillors, opinion leaders and former IRS supervisors. Households that were not in existence between July–August, 2016 when the IRS with Actellic 300 CS happened were excluded from the study. Household survey participants were eligible for selection if they were at least 18 years old and must have been living in the sampled household at the time of spraying. In this study, each randomly sampled parish was considered as a cluster during data collection.

### Variables

The primary dependent variables were household spray status and malaria case numbers. The primary independent variable was the application of IRS. From records, the dependent variables of interest were; total outpatient attendance, outpatient attendance due to confirmed malaria and total malaria cases recorded (clinical & confirmed by microscopy or RDT). The independent variables were calendar time in relation to IRS, rainfall seasonality, name and level of health facility. Data abstraction focused on a 6 months period before and after spraying. In the household survey, the dependent variable was household spray status while independent variables were; socio-demographic characteristics, household characteristics, bed net ownership and use, experience of side effects linked to the previous IRS and willingness to take up the next IRS.

### Sample size and sampling

All the HMIS105 reports from January 2016 to April 2017 at selected health facilities were reviewed. In addition, a total of 159 households were visited and one respondent per household was interviewed. Thirteen key informants were sampled purposively and were interviewed. The sample size for the household survey was calculated using Bennett’s formula of 1991 [21]. The number of clusters were determined considering a design effect of 2.0 and an estimated IRS coverage of 80%, the minimum recommended target for IRS interventions. The investigators anticipated to sample at least 20 households per cluster. The calculated number of clusters (7) was multiplied by the estimated number of households per cluster to obtain the sample size of 140 households which was adjusted for non-response at a rate of 10% as recommended by Centres for Disease Control [22]. After adjusting for non-response, the sample size for household survey was 159 households.

Lira District was purposively sampled because at least 6 months had elapsed after IRS with Actellic 300 CS was conducted. A 6-month interval was considered adequate because evaluation of IRS interventions are normally carried out after 6 months. High volume facilities (i.e., level four health facilities and higher) are expected to have functional laboratories, bigger catchments and to receive ill patients from lower level health facilities. Therefore, they are more likely to give rise to representative data. A two-stage cluster sampling approach was employed to select households. A list of all parishes in the district was obtained from the district population department and using a random number generator, seven parishes were selected with no repeat. Systematic sampling was then applied to select households from each cluster (parish). Random numbers (1–5) were allocated to data collectors to guide them to randomly select the first household per day. Each random number represented the number of households from the village chairperson’s home to be skipped in order to select the first household. The next household was selected after an interval of three households. Sampling of households was proportionate to size and about 20 households were sampled from each cluster.

### Data collection and instruments

Data from medical records was collected using a data abstraction tool and a structured questionnaire was used to collect household survey data. A key informant interview guide was used to collect qualitative data on individual opinions about perceived effect of IRS on malaria morbidity following the spraying intervention.

### Data analysis and management

Malaria morbidity data from HMIS monthly paper reports and household survey data were entered separately in EPI-INFO version 7.2.1.0 and exported to STATA version 13 for analysis. Imputation method was used to fill missing age-group specific data by computing the average of the closest month cells. Responses from key informants were summarized into themes and quotes were extracted and reported verbatim to supplement results from routine surveillance data. Malaria morbidity data was arranged into monthly intervals to form at least twelve data points. The IRS coverage/household spray status was measured as a proportion of households sprayed to total households visited by research assistants. For this study, the focus was on unsprayed households and associated factors. Therefore, a household being unsprayed was considered a positive outcome during data analysis. Factors associated with not spraying were obtained using modified Poisson regression models employing a stepwise elimination method. A Poisson regression model was the preferred analysis approach because the proportion of the outcome of interest (i.e., Household spray status) was high and logistic regression would overestimate the measure of association. A p-value cut-off of 0.2 was considered to select variables for multivariable analysis. To analyse household spray status in the regression model, the option **‘**not sprayed’ was assigned a higher code since it was the outcome of interest. Since medical records review generated count data, the ordinary Poisson regression model was considered appropriate and was fitted to generate percentage point changes (pp) in malaria positivity rate adjusting for seasonality and variations at facility level. Malaria test positivity rate was measured as confirmed malaria cases expressed as a percentage of total malaria cases diagnosed. Wealth index was used as a proxy for social economic status of visited households. It was calculated using data on possession of household items, type of household, means of transport to the health facility, number of meals per day and other dwelling characteristics. Scores were assigned to each of the items considered using Principal Component Analysis (PCA). The sample was divided into 5 quintiles (1–5) representing poorest to richest categories respectively.

## Results

### Background characteristics of the study population

A total of 159 households from 07 parishes in the study district were visited. The age of respondents ranged from 18 to 90 years with a mean of 38.05 years (S.D. ± 16.21). More than a half 57.9% (92/159) of respondents were females and 84.9% (135/159) were married. Half 51.6% (82/159) of the respondents attended primary education and most 64.8% (103/159) were peasants. More than half 66.7% (106/159) of the respondents lived in rural setting. (Table 1). About 90% (143/159) of the households visited owned at least one mosquito bed net (Table 2). The total outpatient attendance was 92,181 before IRS and 79,069 after spraying. Malaria Test Positivity rate (TPR) was 27.0% (4660/17,232) before IRS and 26.7% (3187/11931) after IRS (Table 4).

**Table 1:** Socio-demographic and characteristics of survey participants in Lira District, June 2017

Characteristic	Categories	Frequency, N = 159	Percentage
Age groups	18–24 years	36	22.6
	25 +	123	77.4
Sex	Male	67	42.1
	Female	92	57.9
Marital status	Married	135	84.9
	Not married	24	15.1
Place of residence	Rural	106	66.7
	Urban	53	33.3
Education level	Never attended school	23	14.5
	Primary	82	51.6
	Secondary	27	17.0
	Post-secondary	27	17.0
Occupation of HH head	Peasant	103	64.8
	Small business	21	13.2
	Formal employment	35	22.0
Wealth index	Poorest	42	26.4
	Poor	22	13.8
	Middle	36	22.6
	Rich	29	18.4
	Richest	30	18.9
House type	Temporary	64	40.2
	Semi-permanent	34	21.4
	Permanent	61	38.4
Number of meals per day	One meal	47	29.5
	Two meals	85	53.5
	Three meals	27	16.9

### Coverage of IRS with Actellic 300 CS in Lira District between July–August 2016

Results from the household survey show that 89.3% (142/159) of the households visited were sprayed and about 90% (143/159) households owned at least one bed net (Table 2). Qualitative data also revealed that spraying against mosquitoes had occurred in their communities and most of the households had been sprayed as quoted below.

**Table 2:** Coverage of IRS and characteristics of Households in Lira District between July–August 2016

Factor	Frequency (N = 159)	Percentage
Household was sprayed against mosquitoes		
Yes	142	89.3
No	17	10.7
Household is willing to take up the next round of IRS		
Yes	138	86.8
No	21	13.2
Household members experiences side effects		
Yes	23	14.6
No	124	77.9
Refused to answer	12	7.5
Household owns a mosquito net		
Yes	143	89.9
No	16	10.1

*“I have seen people carrying cans moving house to house to spray houses” (Key Informant (KI), Banya parish)*

*“…not all but most of them were sprayed, the only household that remained in my village had a sick person” (KI, Omito parish).*

### Factors for not spraying and individual household perceptions about the IRS intervention in Lira District

The study found that 13.2% (21/159) of respondents were not willing to take up the next IRS round. Only about 15.0% (23/159) of survey respondents had experienced side effects after IRS (Table 2). Key informants expressed fears of poisoning, unpleasant smells of the insecticide and other health related negative effects after spraying of dwelling structures as expressed in the quotes below.

*“For us in the village, we sleep in grass thatched houses and after eating, they get grass to remove food remains from the teeth, …they pick grass from the roof and this exposes them to the poisonous chemical.” (KI, Orit Parish).*

*“After spraying the household, it takes a very long time for the smell to disappear and when a child touches the wall with wet hands, it can be a poison to the child even.” (KI, Omito Parish).*

At bivariable analysis, respondents who had attended primary education were less likely to live in unsprayed households than respondents who never attended school (PR = 0.21, CI 0.05–0.88, P-value = 0.032). Households headed by formally employed persons were more likely to be unsprayed than those headed by peasants (PR = 5.30, CI 1.89–14.80, P-value < 0.001). The association between socio-demographic factors and IRS spray status are presented in Table 3.

**Table 3:** Association between socio-demographic factors and IRS spray status of households in Lira District, June 2017

Characteristic	Spray status (N = 159)		Crude PR (95% CI)	P-value	Adjusted PR (95% CI)	P-value
	Sprayed n = 142 (%)	Unsprayed n = 17 (%)				
Age group of respondents						
18–24 years	35 (24.6)	1 (5.9)	1.0		1.0	
25 years and above	107 (75.4)	16 (94.1)	4.68 (0.64–34.33)	0.129	2.47 (0.33–14.70)	0.382
Sex						
Male	63 (44.4)	4 (23.5)	1.0		1.0	
Female	79 (55.6)	13 (76.5)	2.36 (0.80–6.96)	0.118	1.72 (0.51–5.75)	0.381
Marital status						
Married	121 (85.2)	14 (82.3)	1.0			
Not married	21 (14.8)	3 (17.7)	1.21 (0.37–3.89)	0.755		
Place of residence						
Rural	97 (68.3)	9 (52.9)	1.0		1.0	
Urban	45 (31.7)	8 (47.1)	1.77 (0.73–4.35)	0.208	0.41 (0.16–1.05)	0.063
Education of household Head						
Never attended	19 (13.4)	4 (23.5)	1.0		1.0	
Primary	79 (55.6)	3 (17.7)	0.21 (0.05–0.88)	0.032	0.18 (0.01–2.47)	0.198
Secondary	24 (16.9)	3 (17.7)	0.63 (0.16–2.6)	0.529	0.36 (0.04–2.95)	0.341
Post-secondary	20 (14.1)	7 (41.1)	1.49 (0.49–4.5)	0.476	1.51 (0.10–22.79)	0.767
Occupation of household head						
Peasant	98 (69.0)	5 (29.4)	1.0		1.0	
Small business	18 (12.7)	3 (17.7)	2.94 (0.75–11.43)	0.119	3.84 (1.20–12.31)	0.024
Formal employment	26 (18.3)	9 (52.9)	5.30 (1.89–14.80)	< 0.001	5.81 (2.72–12.68)	< 0.001

At multivariable analysis, respondents from households headed by formally employed persons and households headed by business owners were more likely than peasants to live in unsprayed households (APR = 5.81, CI 2.72–12.68, P-value < 0.001) and (APR = 3.84, CI 1.20–12.31, P-value = 0.024) after adjusting for age category, place of residence and willingness to take-up the next IRS round. Households of participants who attended post-secondary education were more likely to be unsprayed than for those who never attended formal education (APR = 1.49, CI 0.49–4.50, P-value = 0.76). The details about factors associated with IRS spray status are presented in Table 3.

### Malaria morbidity at outpatient facilities before and after IRS with Actellic 300 CS in Lira District, January 2016 to February 2017

Malaria was responsible for 18.7% (17,232/92,181) of outpatient attendance before IRS with 5.1% (4660/92,181) of the total outpatient attendance testing positive for malaria. The outpatient attendance due to malaria dropped to 15.1% (11,931/79,069) after IRS and the proportion of outpatient attendance due to confirmed malaria had dropped from 5.1% (4660/92,181) to 4.0% (3187/79,069) after IRS intervention (Table 4).

Within 6 months before IRS and considering January, 2016 (i.e., the 6th months before IRS intervention) as a reference month, an increasing trend in malaria burden over time was observed. The highest increase in malaria test positivity rate before spraying was seen in May, June and July, 2016; May (Adj. pp = 0.66, P-value = 0.012), June (Adj. pp = 0.97, P-value < 0.001) and July (Adj. pp = 0.71, P-value = 0.002) controlling for variations at health facility level and seasonality. The percentage point increase in outpatient attendance due to confirmed malaria before IRS was more noticeable in May and June 2016.

After IRS intervention, there was a decline in malaria morbidity (TPR) per unit increase in months. The highest decline was observed 5–7 months after the IRS intervention in January 2017 (Adj. pp = − 0.60, P-value = 0.015), February 2017 (Adj. pp = −1.19, P-value < 0.001) and March, 2017 (Adj. pp = −1.97, P-value = 0.081)  (Table 5).

**Table 4:** Malaria morbidity at outpatient facilities before and after IRS with Actellic 300 CS in Lira District, January 2016 to February 2017

Time (month) in relation to IRS intervention	Total OPD attendance	Total Malaria cases diagnosed	Confirmed malaria cases	Proportion of OPD attendance due to confirmed malaria	Proportion of OPD attendance due to total Malaria	Proportion of total malaria cases confirmed (TPR)
Jan 2016	15,013	2778	627	4.2	18.5	22.6
Feb 2016	12,889	1903	340	2.6	14.8	17.9
Mar 2016	12,659	1727	267	2.1	13.6	15.5
April 2016	13,885	2019	439	3.2	14.5	21.7
May 2016	15,973	3903	1160	7.3	24.4	29.7
June 2016	10,780	2748	1047	9.7	25.5	38.1
July 2016	10,982	2154	780	7.1	19.6	36.2
Before IRS	92,181	17,232	4660	5.1	18.7	27.0
Aug 2016	13,871	1487	776	5.6	10.7	52.2
Sept 2016	13,287	1918	515	3.9	14.4	26.9
Oct 2016	6754	1779	446	6.6	26.3	25.1
Nov 2016	13,912	2136	573	4.1	15.4	26.8
Dec 2016	10,193	1700	395	3.9	16.7	23.2
Jan2017	12,127	1491	319	2.6	12.3	21.4
Feb 2017	8925	1420	163	1.8	15.9	11.5
After IRS	79,069	11,931	3187	4.0	15.1	26.7

**Fig. 1:**
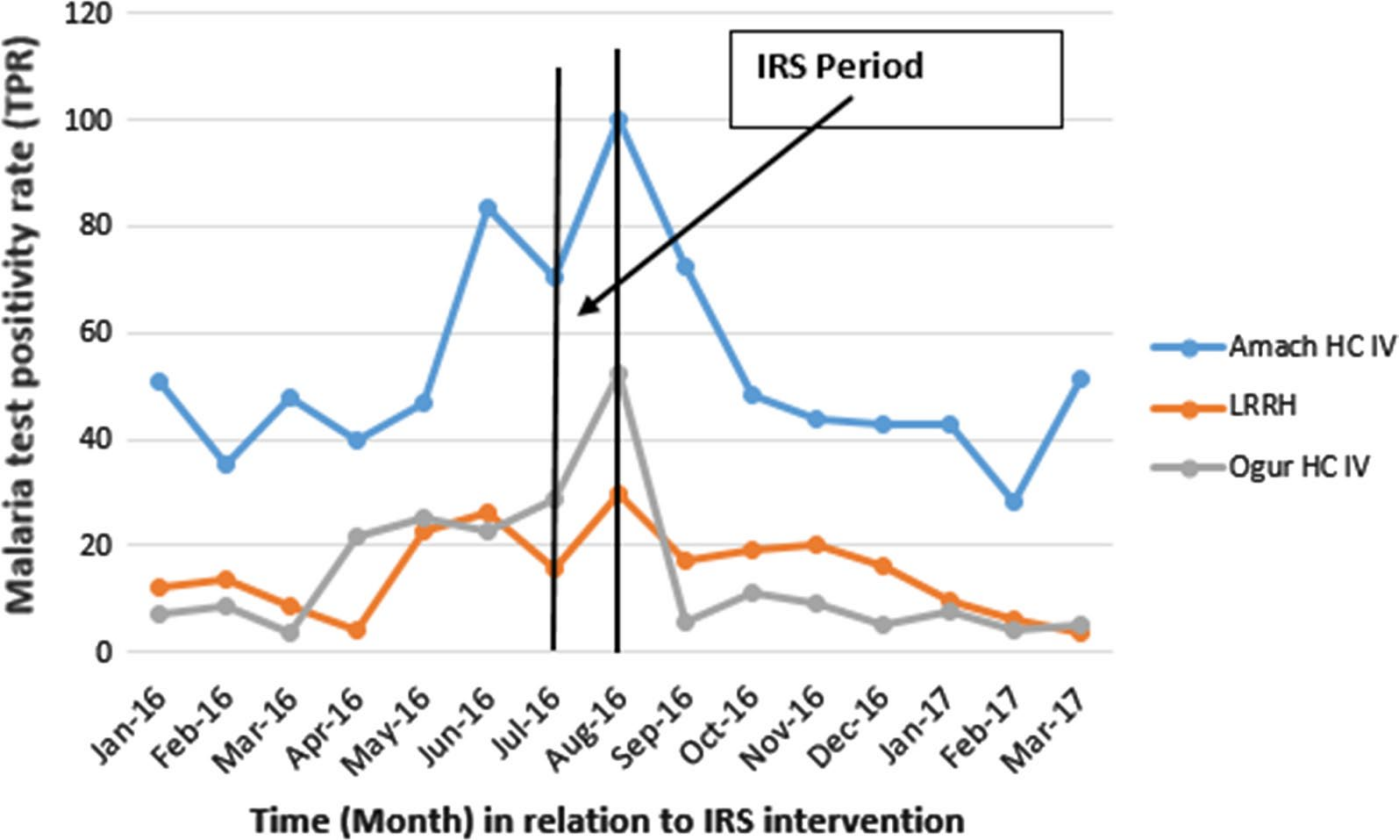
Trends in malaria test positivity rate (TPR) before and after spraying with Actellic 300 CS in Lira District, 2016

Overall, Malaria Test Positivity rate at outpatient facilities was highest in August 2016, the 2nd months of IRS intervention. However, trends in TPR declined after spraying and the decline was consistent for 6 months after spraying across all the health facilities considered (Fig. 1).

**Table 5:** Regression results adjusted for variations at health facility level and seasonality, Jan 2016–Mar 2017

Time in months in relation to IRS with Actellic 300 CS	Adjusted percentage point changes (pp) in confirmed Malaria	95% confidence interval		P-value
Percentage point changes before IRS intervention				
Jan 2016	Ref.			
Feb 2016	− 0.05	− 0.58	0.47	0.836
March 2016	− 1.03	− 1.60	− 0.45	< 0.001
April 2016	− 0.35	− 0.83	0.13	0.153
May 2016	0.66	0.14	1.17	0.012
June 2016	0.97	0.53	1.40	< 0.001
July 2016	0.71	0.27	1.16	0.002
Percentage point changes after IRS intervention				
August (spray month)	Ref			
Sept (1 months after IRS)	− 0.07	− 0.98	0.84	0.878
Oct (2 month after IRS)	− 0.18	− 1.09	0.74	0.702
Nov (3 month after IRS)	− 0.13	− 1.04	0.78	0.781
Dec (4 month after IRS)	− 0.39	− 0.85	0.05	0.085
Jan 17 (5 month after IRS)	− 0.60	− 1.08	− 0.12	0.015
Feb 17 (6 month after IRS)	− 1.19	− 1.79	− 0.60	< 0.001
Mar 17 (7 month after IRS)	− 1.97	− 2.10	0.12	0.081

Adj. pp = percentage point change in outpatient attendance due to confirmed Malaria, adjusted for seasonality and variation at health facility level

## Discussion

This study found that the coverage of IRS with Actellic 300 CS in Lira District was 89.3% and was associated with a prolonged reduction in malaria morbidity trends 6 month after the IRS intervention. A prominent reduction in malaria morbidity was seen after 5–6 months of spraying across all health facilities involved in the study. The reduction in malaria morbidity following IRS has been reported in other studies in Africa. A study on the residual efficacy of Actellic 300 CS in high vector resistance to pyrethroids and carbamates in Zambia reported that IRS was more effective for 5–8 months after spraying [14]. Another study conducted in Benin reported that, IRS with a new organophosphate insecticide formulation, similar to the insecticide being assessed by this study was found to be effective for up to 10 months after spraying [23]. Factors significantly associated with not spraying were; having a formal employment and owning a business.

The observed IRS coverage was higher than 85%, the Uganda Malaria Reduction Strategic Plan target for 2014–2020 [24]. IRS coverage with Actellic 300 CS was found to be lower than the 95% target by Uganda IRS Phase II [25]. The proportion of sprayed households was higher than the 87% reported in Zimbabwe which led to a reduction in malaria incidence by 38% [26]. The coverage of IRS was also higher than that of the previous IRS intervention reported at only 61% [25]. Programmatic reports show that Lira District had experienced a strong resistance against IRS, mostly in Agali and Amach Sub-Counties during the previous IRS round [25]. The observed good coverage of IRS and its effect on malaria case numbers is a sign of progress towards achieving malaria pre-elimination status by 2020.

Households with higher wealth index were less likely to be sprayed. The unsprayed households were likely to belong to formal employers and business people. This could be attributed to gaps in planning for spraying activities which excluded part of the weekend when most of the formal employees and business operators were more likely to be at home to provide access to sprayable structures. This study revealed that in most households that were unsprayed, spray operators either found houses closed or with no eligible person to grant permission to spray.

The lack of knowledge about spraying schedules by locals might also justify the low coverage of IRS among households of formal employees and business operators. A study conducted across sub-Saharan Africa revealed that poorer households were more likely to spray than richer households and this is consistent with findings of this study [27].

Importantly, this study revealed that 90% of the visited households owned mosquito nets. This may also imply that respondents of unsprayed households might have preferred other malaria control interventions, such as bed nets to IRS. A study conducted in Mozambique showed that most respondents preferred bed net use for malaria vector control to IRS [28]. Respondents from unsprayed households also reported that spraying chemicals is harmful and not effective. They also stated that moving items from structures to be sprayed is inconveniencing when a plan to cover all items is not available. Findings from this study are in agreement with a study conducted in Wakiso District, Uganda which indicated that many communities had faced challenges using malaria prevention services due to inconveniences associated with carrying items from the rooms to be sprayed [29]. Similar concerns of harmful effects and ineffectiveness of IRS chemicals were reported in a Mexican study [30]. The fear that buyers would reject crops stored in sprayed structures might have also contributed to the proportion of unsprayed household since most of the respondents were peasants.

Most of the respondents, 79% had observed a reduction in malaria related sickness in their households or communities after spraying. The reduction in malaria among household members after spraying is comparable to another study in Northern Uganda which found that malaria morbidity was lower in sprayed communities and was estimated at 37% than in non-IRS communities 49.8% [31]. A study by Kigozi et al. conducted in Gomba district, Central Uganda, reported similar findings [32]. This is also in agreement with Kim’s finding from various countries which indicated a risk reduction in malaria morbidity of over 60% after IRS [33].

Analysis of malaria data at outpatient facilities revealed that IRS with Actellic 300 CS was associated with a prolonged reduction in malaria morbidity trends across all health facilities involved in the study. The significant decrease in malaria burden after spraying had coincided with a period when malaria burden was expected to be low due to seasonality variations. However, the coincidence is unlikely to justify that the reduction was due to seasonal effects since the trend consistently remained lower including the periods when an upsurge in malaria cases was expected. The decreasing impact in malaria morbidity after spraying supports the reported prolonged effective action of Actellic 300 CS against malaria vectors [14]. The outpatient attendance due to total malaria was 15–18%, a rate lower than the 30–50% national burden [4]. Other studies in Tanzania, Zambia, Zanzibar, Zimbabwe and Benin have showed similar trends in malaria burden after spraying interventions with an organophosphate [14, 23, 26, 34, 35].

### Study limitations

This study assumed that the impact of other malaria control interventions in place was normally distributed across all health facilities and households. The assumption was based on the fact that there were no new malaria control interventions in place during the spray intervention and study period. Furthermore, the trends in malaria morbidity were assessed basing on secondary data, which is more likely to be biased by health seeking behaviours and reporting incompleteness. This study did not analyse data from lower level facilities and also did not use a control group in this study. However, the baseline data for the 6 months period before the IRS intervention was used as a proxy for the malaria situation at baseline had the IRS intervention not taken place at the time it did.

### Generalizability of results

In order to improve the study power and generalizability of results, this study collected data from high volume health facilities, which also receive ill patients from lower level health facilities through a referral system. The health facilities considered were representative of the entire district since they were selected from all the three health sub-districts. Therefore, the findings of this study may be generalizable to the entire district. A household survey was conducted to supplement secondary data analysis and to improve the study power through methods triangulation.

## Conclusion

This study found that coverage of IRS with Actellic 300 CS in Lira District was high. Malaria morbidity trends at outpatient facilities were consistently lower after the IRS intervention. The decreasing effect of IRS with Actellic 300 CS on malaria morbidity was more prominent 5–6 months after IRS.

### Recommendations

- Stakeholders involved in planning and implementation of IRS projects should extend the spraying activities to cover all weekdays including weekends and public holidays. This may help households of formally employed persons and business owners to benefit from IRS intervention.
- An enforcement of community awareness campaigns to inform people about the benefits of spraying and all necessary preparations required for safer spraying.
- This study recommends that further studies be conducted to compare the effect of Actellic 300 CS with other insecticide classes in similar settings.
- This study also recommends further studies to assess the impact of IRS with Actellic 300 CS using a longer study period before and after the intervention.

## Abbreviations

APR: adjusted prevalence ratio
CI: confidence interval
CS: capsulated suspension
DHO: District Health Office
HMIS: Health Management Information Systems
IRS: indoor residual spraying
KI: key informant
PAG: Pentecostal Assemblies of God
PCA: Principal Component Analysis
PMI: President’s Malaria Initiative
PNFPs: private not for profit
pp: percentage point change
PR: prevalence ratio
RDT: rapid diagnostic test
TPR: test positivity rate
WHOPES: World Health Organization Pesticides Evaluation Scheme
